# Depression and Anxiety Among Dentists: A Systematic Review and Meta‐Analysis

**DOI:** 10.1002/hsr2.70786

**Published:** 2025-05-05

**Authors:** Zrnka Kovačić Petrović, Tina Peraica, Mirta Blažev, Vesna Barac Furtinger, Dragica Kozarić‐Kovačić

**Affiliations:** ^1^ Department of Psychiatry and Psychological Medicine University of Zagreb School of Medicine Zagreb Croatia; ^2^ Department of Addiction University Hospital Vrapče Zagreb Croatia; ^3^ Department of Psychiatry, Referral Center for Stress‐Related Disorders of the Ministry of Health University Hospital Dubrava Zagreb Croatia; ^4^ Department of Forensic Sciences University of Split Split Croatia; ^5^ Ivo Pilar Institute of Social Sciences Zagreb Croatia; ^6^ Dentist Office Vesna Barac Furtinger Zagreb Croatia

**Keywords:** anxiety, dentists, depression, gender, prevalence

## Abstract

**Background and Aims:**

Many studies investigated the prevalence and severity of depression and anxiety among dentists. This systematic review aimed to determine: (i) the prevalence and severity of depression and anxiety symptoms, (ii) the prevalence rates of depression and anxiety before and during the COVID‐19 pandemic, and (iii) gender difference in prevalence of depression and anxiety among dentists.

**Methods:**

Eligible articles on depression and anxiety in dentists were systematically searched for in PubMed and Scopus databases from September 2023 to October 2023 according to the Preferred Reporting Items for Systematic Review and Meta‐Analysis protocol. We assessed the methodological quality of the studies using the Newcastle–Ottawa Quality Assessment checklist adapted for cross‐sectional studies. Statistical heterogeneity across the studies was evaluated using Cochran's *Q* test and *I* ^2^ statistic. The prevalence rates of depression and anxiety were calculated using the random‐effect model with the Restricted Maximum‐Likelihood estimator. Of 3762 searched articles, 33 articles were analyzed.

**Results:**

The prevalence rates of depression and anxiety symptoms among dentists were 42% and 44%, respectively. The prevalence rates of mild, moderate, and severe or extremely severe depression were 20%, 18%, and 8%, respectively. For mild, moderate, and severe or extremely severe anxiety, the respective prevalence rates were 21%, 18%, and 11%. We did not find evidence to suggest differences in depression or anxiety prevalence rates between the periods before and during COVID‐19. In comparison with men, women showed approximately 27% higher risk of experiencing depression and 24% higher risk of experiencing anxiety.

**Conclusion:**

Equally high levels of depression and anxiety in dentists were found both before and during the COVID‐19 pandemic, with a significant percentage of moderate to severe depression and anxiety. Female dentists reported a higher prevalence of depression and anxiety symptoms than their male colleagues.

## 1 Introduction

The symptoms of depression and anxiety in dentists may be considered significant indicators of their work‐related psychological distress [1, 2]. Studies have found that even during their studies, future dentists are faced with increased psychological stress leading to depression and anxiety [1].

During the coronavirus disease 2019 (COVID‐19) pandemic, containment measures, such as lockdown and other restrictions, generated stress, anxiety, depression, and exacerbation of pre‐existing diseases in entire population, including dentists, who were additionally affected due to work‐related risks. The stress among dentists during the COVID‐19 pandemic influenced their burnout, anxiety, and workload [3]. The most pronounced psychological effects were the fear of infection by patients, anxiety, and stress [4]. Research identified increased anxiety levels in dental professionals, especially in younger and female professionals [5], and fear of COVID‐19, anxiety, sadness, concern, and anger [6].

Although considerable attention has been focused on anxiety [6, 7, 8, 9, 10, 11, 12, 13], depression [14, 15, 16, 17, 18], and depression and anxiety [19, 20, 21, 22, 23, 24, 25, 26, 27, 28, 29, 30, 31, 32, 33, 34] in dentists, a review of research on dentist's mental health (anxiety and depression) shows a lack of consistency with regard to operational definitions of the terms and the use of different research methods.

The rationale for this review was that despite the evidence indicating impaired mental health and psychological distress related to depression and anxiety in dentists before and during the pandemic period, research outcomes appear heterogeneous because of (i) different operational definitions of determinants of mental health (depression and anxiety), (ii) different study designs, and (iii) different healthcare systems.

So far, there has been no systematic evidence about depression and anxiety among dentists. The first aim of this systematic review was to determine the prevalence of (i) depression and (ii) anxiety and (iii) the severity of depression and anxiety (mild, moderate, severe or extremely severe) among dentists. The second aim was to determine the moderating role of time period (studies conducted before and during the COVID‐19 pandemic) on the prevalence rates of depression and anxiety among dentists. The third aim was to determine possible gender differences in the prevalence of (i) depression and (ii) anxiety. Finally, we assessed the methodological quality of the studies analyzed in this review to discriminate between weak and strong evidence.

It is important to direct appropriate prevention and treatment interventions targeting dentists during their everyday practice and prolonged pandemic stress to reduce negative impacts on their mental health. Hence, the awareness of dentist's mental well‐being during their education and at workplace should be increased [35].

## 2 Methods

### 2.1 Study Identification

**Figure 1:**
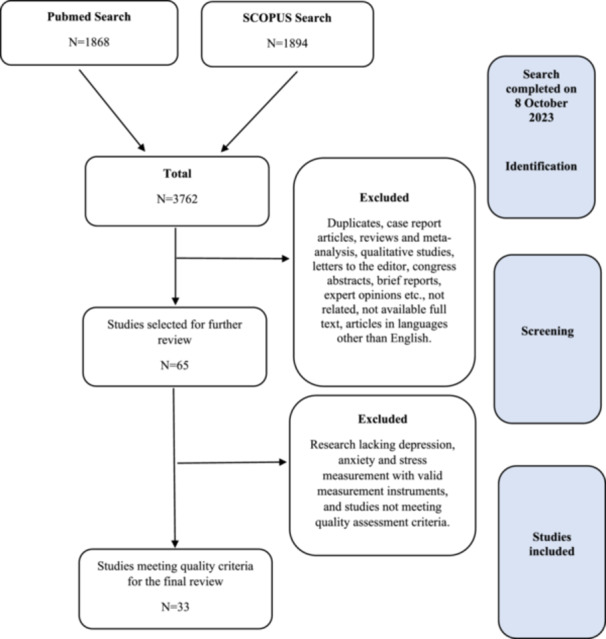
PRISMA 2020 flow diagram for the present review.

We used a structured data format based on the principles of systematic reviews according to the Preferred Reporting Items for Systematic Review and Meta‐Analysis (PRISMA) protocol (Figure 1) [36]. To identify the relevant studies, we searched PubMed and Scopus databases using the following Medical Subject Headings (MeSH) terms as keywords: “dentists,” “mental health symptoms,” “depression,” and “anxiety.” The search was performed from September 2023 to October 2023 in three steps. First, a relevant article was selected by evaluating the title. In the second step, abstracts of relevant articles were read. The third step included reading the complete text of the articles selected in the previous steps. Thus, we obtained a set of articles meeting the inclusion criteria for this systematic review and meta‐analysis.

Much of the identified literature originates from the USA, UK, Turkey, Brazil, Iran, Iraq, Saudi Arabia, and other Middle East region or European countries (Table 1).

**Table 1:** Characteristics of studies on depression and anxiety among dentists included in the meta‐analysis.

Authors	Country	Participants (dentists) gender	Objectives and design	Scales	Prevalence scores	Overall quality score
El‐ Gammal et al. [20]	Egypt, Algeria, Iraq, Jordan, Libya, Palestine, Saudi Arabia, Sudan, Syria, Tunisia, Yemen	Total 4845 health care workers (physicians, nurses, pharmacists and dentists). 605 dentists from 11 different countries 42.5% male 57.5 female	Depression, anxiety and stress levels during COVID‐19 Cross‐sectional design	DASS‐21	Depression 70.9% Anxiety 70.9%	7 Good
Banakar et al. [34]	Iran	638 dental care providers 60.2% male 39.8% female	Mental health outcomes (focusing on insomnia, anxiety, depression, and posttraumatic stress disorder) during COVID‐19 Cross‐sectional design	HADS	Anxiety 40.8% Depression 54.9%	7 Good
Lima et al. [23]	Brazil	404 orthodontists 35.9% male 64.1% female	Depression, anxiety, insomnia and distress during COVID‐19 Cross‐sectional design	PHQ‐9 GAD	Depression 62.4% Anxiety 62.6%	7 Good
Al‐Rawi et al. [30]	Iraq	269 dentists 17.5% male 82.5% female	Depression, anxiety, and stress during COVID‐19 Cross‐sectional design	DASS‐21 PHQ‐9	Any grade of depression 40% Any grade of anxiety 48% Severe/extremely severe depression 10.4% Severe/extremely severe anxiety 19.3%	7 Good
Mohamed Asif et al. [1]	Saudi Arabia	246 dentists 56.33% male 43.67% female	Anxiety and depression among dentists Cross‐sectional design	SAS SDS	Severe anxiety among male dentist 71.74% and moderately depression 69.5% Severe anxiety among female dentist 84.11% and moderately depression 67.29%	7 Good
De Araujo et al. [13]	Brazil	2106 dentists 74.1% male 35.9% female	Stress and anxiety during COVID‐19 Cross‐sectional design	DASS‐21	Anxiety has 74.6% of male, and 48.3% female. Female participants had 63% lower chance of reporting anxiety than males.	7 Good
Eldridge et al. [19]	USA	8902 dental healthcare workers. 2.170 dentists 59.9% male 38.9% female	Anxiety and depression during COVID‐19 Cross‐sectional design	PHQ‐4	Anxiety symptom rates peaked in November 2020 (17% of dentists) and declined to 12% in May 2021. Depression symptom rates were highest in December 2020 (10% of dentists) and declined to 8% in May 2021	7 Good
Li et al. [22]	China	256 dentists 30.1% male 69.9% female	Depression, anxiety, and stress during COVID‐19 Cross‐sectional design	DASS‐42	Depression 33.2% Anxiety 37.1%	7 Good
Alencar et al. [25]	Brazil	998 dentists 27.3% male 72.7% female	Depression, anxiety, and stress during COVID‐19 Cross‐sectional design	DASS‐21	Depression 47.3% Anxiety 46.3%	7 Good
Al‑Amad and Hussein [10]	19 different countries Bahrain, Canada, Egypt, Germany, India, Italy, Jordan, Kuwait, Malaysia, Oman, Palestine, Poland, Qatar, Saudi Arabia, Syria, Turkey, United Arab Emirates, United Kingdom, United States of America	403 dentists 29.9% male 70.1% female	Anxiety during COVID‐19 Cross‐sectional design	GAD‐7	Dentists ‐ minimal anxiety 35.8%; Mild anxiety 34.9%; Moderate anxiety 15.1%; Severe anxiety 14.2% Dental specialists ‐ minimal anxiety 37.3%; Mild anxiety 35.6%; Moderate anxiety 13.6%; Severe anxiety 13.6% Male ‐ minimal anxiety 50.0%; Mild anxiety 30.2%; Moderate anxiety 11.2%; Severe anxiety 8.6% Female ‐ minimal anxiety 26.7%; Mild anxiety 36.5%; Moderate anxiety 18.8%; Severe anxiety 18.1%	7 Good
Arias‐Vasquez and Espinoza‐Salcedo [37]	Northern Peru	310 dentists No data for gender	Depression, anxiety, and stress in dentists in times of COVID‐19. Cross‐sectional design	DASS‐21	Anxiety 35.05%, Depression 29.21% Mild depression 45.88% Moderate anxiety 40.20%.	7 Good
Arslan et al. [33]	Turkey	671 healthcare professionals 45 dentists 44.6% male 55.4% female	Anxiety, depression levels, and psychological resilience among medical doctors and dentists. Cross‐sectional design	HAD‐A HAD‐D	Depression: 46.7% Anxiety: 31.1% Depression and anxiety scores were higher among women.	7 Good
Bellini et al. [12]	Italy	1109 dentists 70.4% male 29.6% female	Fear and anxiety during COVID‐19 Cross‐sectional design	GAD‐7	Intense anxiety 14.5%, light anxiety 33.1%, moderate anxiety 22.6%. Intense concern 23.6%, light concern, 27.6%, moderate concern 30.0%.	7 Good
Campos et al. [38]	Brazil	1609 healthcare workers 341 dentists No data for gender	Depression, anxiety and stress during COVID‐19 Cross‐sectional design	DASS‐21	Depression 57.2% Anxiety 43.1%	
Chen and Li [39]	China	808 dental medical staff 558 dentists 45.5% male 54.5% female	Prevalence and influencing factors of anxiety, depression, perceived stress, and acute stress disorder during COVID‐19. Cross‐sectional design	GAD‐7 PHQ‐9	Depression 46.4% Anxiety 36.3% Individuals with a past medical history reported experiencing more anxiety and depression.	7 Good
Humphris et al. [16]	UK, Scotland	110 dental trainees 218 dentists 21% male and 79% female dental trainees 18% male and 81% female dentists	Depression, stress, and burnout during COVID‐19 Cross‐sectional design	PHQ‐2	Depression 27%	7 Good
Mekhemar et al. [24]	German	732 dentists 40% male 59.7% female 0.3% third gender	Depression, stress, and burnout during COVID‐19 Cross‐sectional design	DASS‐21	Depression 43.3% Severe/extremely severe depression 14.4% Anxiety 30.6% Severe/extremely severe anxiety 11.8%	7 Good
Ranka and Ranka [28]	UK	124 dentists No data for gender	Anxiety, depression, and stress symptoms during COVID‐19 Cross‐sectional design	PHQ‐4	Anxiety 71% Depression 60%	7 Good
Salehiniya and Abbaszadeh [29]	Iran	320 dentists 53.8% male 46.2% female	Anxiety and mental health disorder during COVID‐19 Cross‐sectional design	GHQ‐28	Mild anxiety 32.5% Moderate anxiety 10% Severe anxiety 0% Mild depression 6.2% Moderate depression 2.5% Severe depression 1.3%	7 Good
Sancak et al. [11]	Turkey	1249 physicians 46 dentists	Anxiety and other contributing variables during COVID‐19 Cross‐sectional design	HADS	Depression 34.8% Anxiety 43.5%	7 Good
Sarapultseva et al. [31]	Russia	128 dental healthcare workers 43 dentists 21.1% male 78.9% female	Depression, anxiety, and PTSD during COVID‐19 Cross‐sectional design	DASS‐21	Depression (mild to extremely severe) 20.3% Anxiety (mild to extremely severe) 24.2%	7 Good
Tao et al. [40]	China	969 dentists 32.0% male 68.0% female	Depression, stress, anxiety, and PTSD during COVID‐19 Cross‐sectional design	PHQ‐9 GAD‐7	Depression 13.8% Anxiety 7.1%	7 Good
Consolo et al. [7]	Italy	356 dentists 60.4% male 39.6% female	Practical and emotional consequences of COVID‐19 emergence on daily clinical practice Cross‐sectional design	GAD‐7	42.7% minimal anxiety, 33.3% mild anxiety, 15.2% moderate anxiety, 8.7% severe anxiety.	7 Good
Estrich et al. [21]	USA	2.135 dentists 60.6% male 39.4% female	COVID‐19‐associated symptoms, depression, anxiety, and physical health conditions Cross‐sectional design	PHQ‐4	Depression 8.6% Anxiety 19.5%	7 Good
Martina et al. [8]	Italy	349 dentists 50.1% male 49.9% female	Anxiety, fear, and distress during COVID‐19 Cross‐sectional design	PHQ‐4	Moderate/high distress (anxiety) 22% No/low distress (anxiety) 78% Fear to infect familiars 45.8% Fear of die 23.5% Fear to be discriminated 8.9%	7 Good
Chohan et al. [14]	USA	540 pediatric dentists 47% male 53% female	Occupational burnout and/or depression among US pediatric dentists Cross‐sectional design	PHQ‐9	Depression 7.2%	7 Good
Yilmaz and Onem Ozbilen [9]	Turkey	209 orthodontists 29.8% male 70.2% female	General knowledge, emergencies, personal precautions, and avoided behaviors and anxiety levels during COVID‐19 Cross‐sectional design	GAD‐7	Anxiety 16.7%	7 Good
Prasad et al. [26]	India	242 dentists 66.4% male 33.6% female	Prevalence of anxiety and depression Cross‐sectional design	SAS SDS	Anxiety 44.4% Depression 36.9%	7 Good
Song et al. [32]	South Korea	231 dentists 68.0% male 32.0% female	Correlation of occupational stress with psychosocial stress, depression, anxiety, and sleep among dentists in Korea Cross‐sectional design	CES‐D STAI	Depression 43.7% Anxiety 13.0%	7 Good
Huri et al. [17]	Turkey	337 dentists 51.92% male 48.07% female	Association between depressive symptoms and burnout among Turkish dentists Cross‐sectional design	BDI	Depression mild 22.4% Depression moderate 29.3% Depression severe 22.2%	7 Good
Rahshenas et al. [27]	Iran	80 dentists 28.7% male 21.2% female 31.3% control male 18.8% control female	The effect of art painting in reducing stress, anxiety and depression in dentists Cross‐sectional design	DASS‐42	Depression 75% Anxiety 57.5%	7 Good
Ahola and Hakanen [15]	Finland	2555 dentists 74.0% male 26.0% female	Whether burnout mediates the association between job strain and depressive symptoms Cross‐sectional design	BDI	Depression 23%	7 Good
Mathias et al. [18]	USA	560 dentists 50.7% male 48.9% female 0.4% not specified	Depression in dentists and to determine if sex and dental specialty were correlated with depression in dentists Cross‐sectional design	SDS	Depression 9% 15% of depressed dentists were receiving treatment Female pediatric dentists and periodontists were more depressed than their male counterparts	7 Good

*Note:* Although other valid measuring instruments for stress, burnout, posttraumatic stress disorder (PTSD), and insomnia were used in some of the included studies, they were not listed, as well as the prevalence scores of stress, burnout, PTSD, and insomnia considering the aim of this paper.

Abbreviations: BDI, Beck Depression Inventory; CES‐D, Center for Epidemiologic Studies Depression Scale; DASS‐21, Depression Anxiety and Stress Scale‐21; DASS‐42, Depression Anxiety and Stress Scale‐42; GAD, Generalized Anxiety Disorder Scale; GAD‐7, General Anxiety Disorder Scale‐7; GHQ‐28, General Health Questionnaire 28; HADS, Hospital Anxiety and Depression Scale; PHQ‐4, Patient Health Questionnaire 2; PHQ‐4, Patient Health Questionnaire 4; PHQ‐9, Patient Health Questionnaire 9; SAS, Zung Self‐Rating Anxiety Scale; SDS, Zung Self‐Rating Depression Scale; STAI, State‐Trait Anxiety Index.

### 2.2 Inclusion and Exclusion Criteria

The inclusion criteria were as follows: manuscripts published from January 1, 2000 to October 8, 2023; dentists as one of the studied groups; evaluation of depression and anxiety symptoms; use of valid measurement instruments for depression and anxiety (Depression Anxiety and Stress Scale‐21 (DASS‐21), Depression Anxiety and Stress Scale‐42 (DASS‐42), Patient Health Questionnaire 9 (PHQ‐9), Patient Health Questionnaire 4 (PHQ‐4), Patient Health Questionnaire 2 (PHQ‐4), Hospital Anxiety and Depression Scale (HADS), Generalized Anxiety Disorder Scale (GAD), General Anxiety Disorder Scale‐7 (GAD‐7), Zung Self‐Rating Anxiety Scale (SAS), Zung Self‐Rating Depression Scale (SDS), Beck Depression Inventory (BDI), General Health Questionnaire – 28 (GHQ‐28), Center for Epidemiologic Studies Depression Scale (CES‐D), and State‐Trait Anxiety Index (STAI)); original articles with cross‐sectional design; and studies published in English language.

The following exclusion criteria were applied: evaluation of mental health disorders such as behavioral and emotional disorders, bipolar affective disorders, dissociation, dissociative disorders, obsessive‐compulsive disorders, psychotic disorders, and paranoia; studies on healthcare professionals excluding dentists; studies on dental staff and workers other than dentists; articles in languages other than English, studies examining the effect of other pandemics on dentist's mental health; case report articles, reviews and meta‐analysis, qualitative studies, letters to the editor, congress abstracts, brief reports, expert opinions, etc.; studies estimating the prevalence or incidence rates of occupational diseases in dentists without explanatory factors.

Three aspects were checked for quality assessment of selected articles: (1) methodology, (2) accuracy, and (3) external validity. We used the Newcastle–Ottawa Quality Assessment checklist adapted for cross‐sectional studies for evaluating the quality of the articles [41].

Of 65 identified eligible studies, 19 were excluded for the absence of specific data on depression or anxiety in dentists (with some studies providing only cumulative data for healthcare workers), the use of novalidated instruments, or the presence of the studies by the same authors with the same data set. This resulted in 46 studies for further analysis. Of these 46 studies, 13 were further excluded due to insufficient data (e.g., only average scores were reported, or there were no separate data for depression and anxiety as they were presented in a merged format). The final sample, thus, consisted of a total of 33 studies eligible for inclusion in the meta‐analysis (Table 1).

The four reviewers reached a consensus on including 33 studies in this review after excluding manuscripts according to the inclusion and exclusion criteria. Only studies that all reviewers unequivocally considered suitable for analysis were included.

Of these 33 studies, 28 studies provided data on overall depression and 27 studies provided data on anxiety symptoms, along with 11 studies reporting the severity of these symptoms and gender differences. The remaining studies focused only on one of these aspects. Consequently, a different number of studies was used to answer different research questions (Table 1).

Initially, our data set included a limited number of studies with gender‐specific data: six for depression and eight for anxiety. To address this, we proactively contacted the authors of all 65 initially eligible studies and asked them for additional data, specifically, data on depression and anxiety if missing, data on dentists excluded from healthcare workers, or additional information on gender prevalence of depression and anxiety.

Eight authors replied. Unfortunately, three of these responses were not positive. One author declined to provide the data, another had retired and no longer had access to the data, and the third indicated that the sample size of dentists was too small (less than five) to give meaningful results. However, the remaining five responses provided valuable additional data, enabling us to improve the sample size in our analysis. As a result of this, we were able to include more studies that addressed gender differences. Consequently, our data set expanded to include 11 studies for gender differences in depression and 13 studies for gender differences in anxiety.

### 2.3 Statistical Analysis

The statistical analysis focused on estimating the overall prevalence rates of depression and anxiety symptoms, as well as subgroup differences based on the time period (pre‐COVID and during‐COVID) and gender. Analyses were stratified by severity of depression and anxiety symptoms (mild, moderate, severe, and extremely severe). The primary (pre‐specified) analyses included the overall prevalence rates and the subgroup comparisons by time period and gender. Exploratory analyses were conducted to investigate patterns in publication bias and heterogeneity. In our meta‐analytic approach, we followed the framework provided by Borenstein et al. [42].

Depression and anxiety were assessed as dichotomous variables, representing the presence or absence of symptoms for both depression and anxiety. Prevalence and severity levels were analyzed using proportional meta‐analysis. To investigate the moderating role of time period (studies conducted before and during the COVID‐19 pandemic) on the prevalence rates of anxiety and depression symptoms among dentists, we employed proportional meta‐analysis with subgroups (pre‐COVID and during‐COVID). Additionally, we examined gender differences in prevalence of depression and anxiety symptoms using risk ratio (RR) meta‐analysis.

Statistical heterogeneity across studies was evaluated using both the Cochran's Q test (which tests the null hypothesis of homogeneity across studies) and I^2^ statistic (which quantifies the percentage of variability in effect estimates attributable to heterogeneity rather than chance). The interpretation of heterogeneity followed the guidelines described by Higgins et al. [43]. Given the observed heterogeneity of the studies (I² > 75%), we employed a random‐effects model with Restricted Maximum‐Likelihood estimator to account for variability in underlying study populations. For the pooled estimates of prevalence rates, we calculated raw proportion rates, while for gender differences, we calculated log‐transformed risk ratios and then transformed them back into risk ratios (RR) for easier interpretability. For each estimate, we calculated 95% confidence intervals (CI) to provide a measure of precision.

To assess potential publication bias, we performed a series of analyses. Funnel plots were visually inspected to detect asymmetry, which could indicate bias. Quantitative tests for funnel plot asymmetry included the Rank correlation test and the Regression test. Additionally, the Fail‐safe N analysis was conducted to evaluate the robustness of the results against the inclusion of unpublished or nonsignificant studies.

All statistical tests were conducted using a two‐sided approach, with an a‐priori significance level set at 0.05. All analyses were performed using the R‐based program jamovi [44]; Version 2.3.

## 3 Results

Due to the heterogeneity of the studies (Cochran's Q test *p* < 0.001; *I* ^2^ > 75%) [43], a random effect model with Restricted Maximum‐Likelihood estimator was applied for all subsequent analyses (Table 2).

**Table 2:** Meta‐analysis of depression (*k* = 28) and anxiety symptoms prevalence (*k* = 27) and severity of these symptoms among dentists: subgroup analysis by time period (pre/during COVID) and gender differences.

Intercept	Raw proportion	SE	z	*p*	95% CI	*I* ^2^ (%)	Q	df	*p*
Depression	0.423	0.05	9.40^†^	< 0.001	0.34, 0.51	99.7	40,361.1^†^	27	< 0.001
Anxiety	0.435	0.04	10.1^†^	< 0.001	0.35, 0.52	99.6	29,013.6^†^	26	< 0.001
Depression ‐ severity									
Mild	0.199	0.03	6.59^†^	< 0.001	0.14, 0.26	98.2	426.1^†^	16	< 0.001
Moderate	0.177	0.04	4.80^†^	< 0.001	0.10, 0.25	99.1	773.3^†^	16	< 0.001
Severe and extr. severe	0.078	0.02	3.59^†^	< 0.001	0.04, 0.12	99.8	664.9^†^	16	< 0.001
Anxiety ‐ severity									
Mild	0.210	0.03	6.77^†^	< 0.001	0.15, 0.27	97.7	620.5^†^	14	< 0.001
Moderate	0.183	0.05	4.05^†^	< 0.001	0.10, 0.27	99.2	830.4^†^	14	< 0.001
Severe and extr. severe	0.110	0.03	3.88^†^	< 0.001	0.05, 0.17	99.3	792.5^†^	14	< 0.001
Pre/during COVID									
Pre COVID‐D	0.494	0.08	5.87^†^	< 0.001	0.33, 0.66	99.6	14,687.4^†^	27	< 0.001
During COVID‐D	0.395	0.05	7.4^†^	< 0.001	0.29, 0.50				
Difference	0.099	0.10	1.00	0.32	−0.10, 0.30				
Pre COVID‐A	0.537	0.11	4.8^†^	< 0.001	0.32, 0.76	99.4	6428.0^†^	26	< 0.001
During COVID‐A	0.417	0.05	8.93^†^	< 0.001	0.33, 0.51				
Difference	0.120	0.12	0.98	0.33	−0.12, 0.36				
Gender differences									
	Log risk ratio	SE	*z*	*p*	95% CI	*I* ^2^ (%)	Q	df	*p*
Women‐D	0.236	0.07	3.26^†^	0.001	0.09, 0.38	73.3	55.2^†^	10	< 0.001
Women‐A	0.214	0.10	2.24^†^	0.025	0.03, 0.40	93.0	170.6^†^	12	< 0.001

*Note:* D‐Depression; A‐anxiety; Severe and extr. severe, severe and extremely severe; Estimator: restricted maximum‐likelihood; Log risk ratio transformed into risk ratio (*RR*) values: Women‐D *RR* = 1.266, 95% CI [1.10, 1.46]; Women‐A *RR* = 1.239, 95% CI [1.03, 1.50]. Although other valid measuring instruments for stress, burnout, posttraumatic stress disorder (PTSD), and insomnia were used in some of the included studies, they were not listed, as well as the prevalence scores of stress, burnout, PTSD, and insomnia considering the aim of this paper. ^†^ *p* < 0.05.

### 3.1 The Prevalence of Depression and Anxiety Symptoms and the Severity of Depression and Anxiety Symptoms Among Dentists

**Figure 2:**
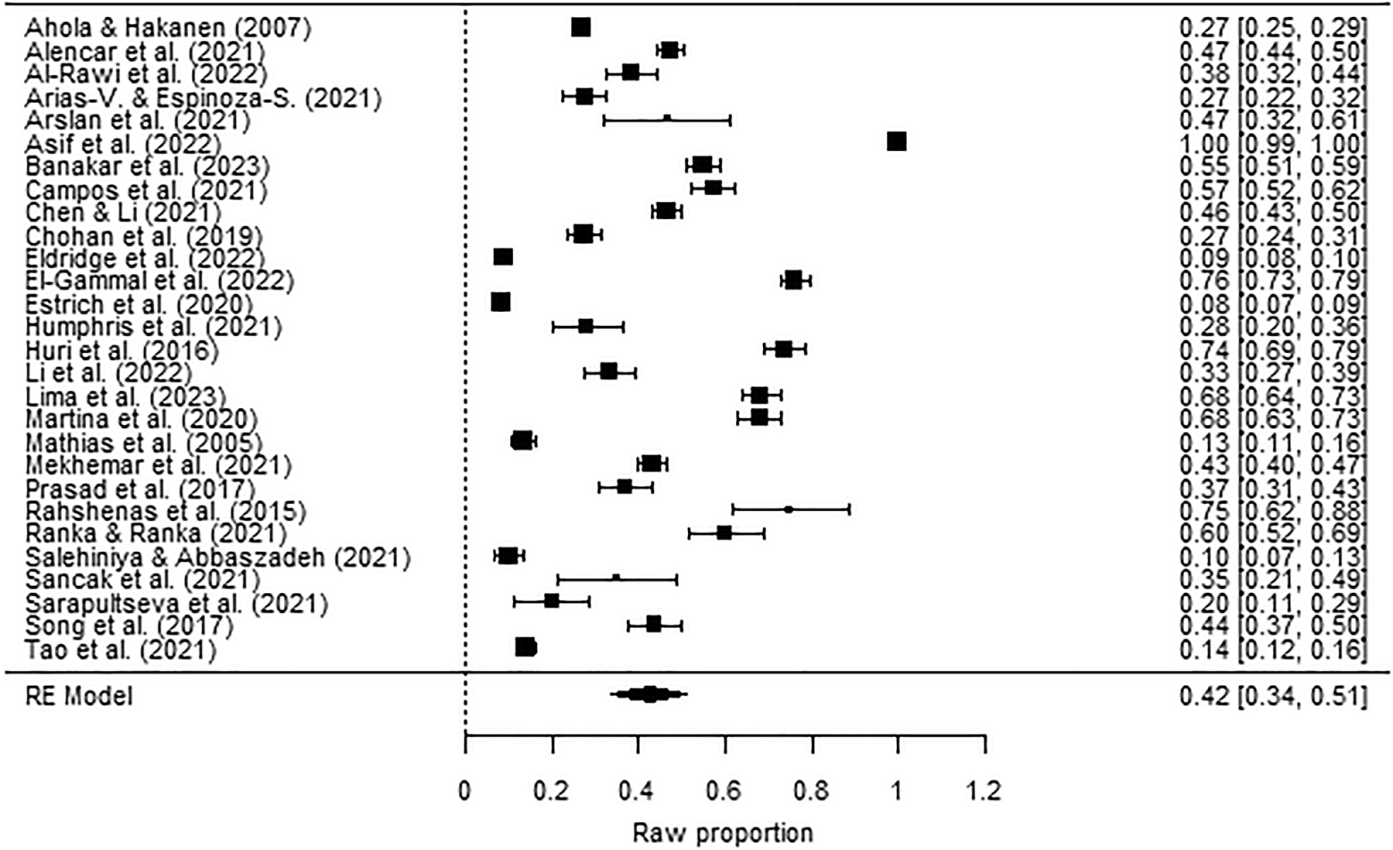
Forest plot–prevalence of depression symptoms among dentists (*k* = 28). The full surnames of the authors Arias‐Vásquez and Espinoza‐Salcedo are shortened in the figure due to technical reasons.

**Figure 3:**
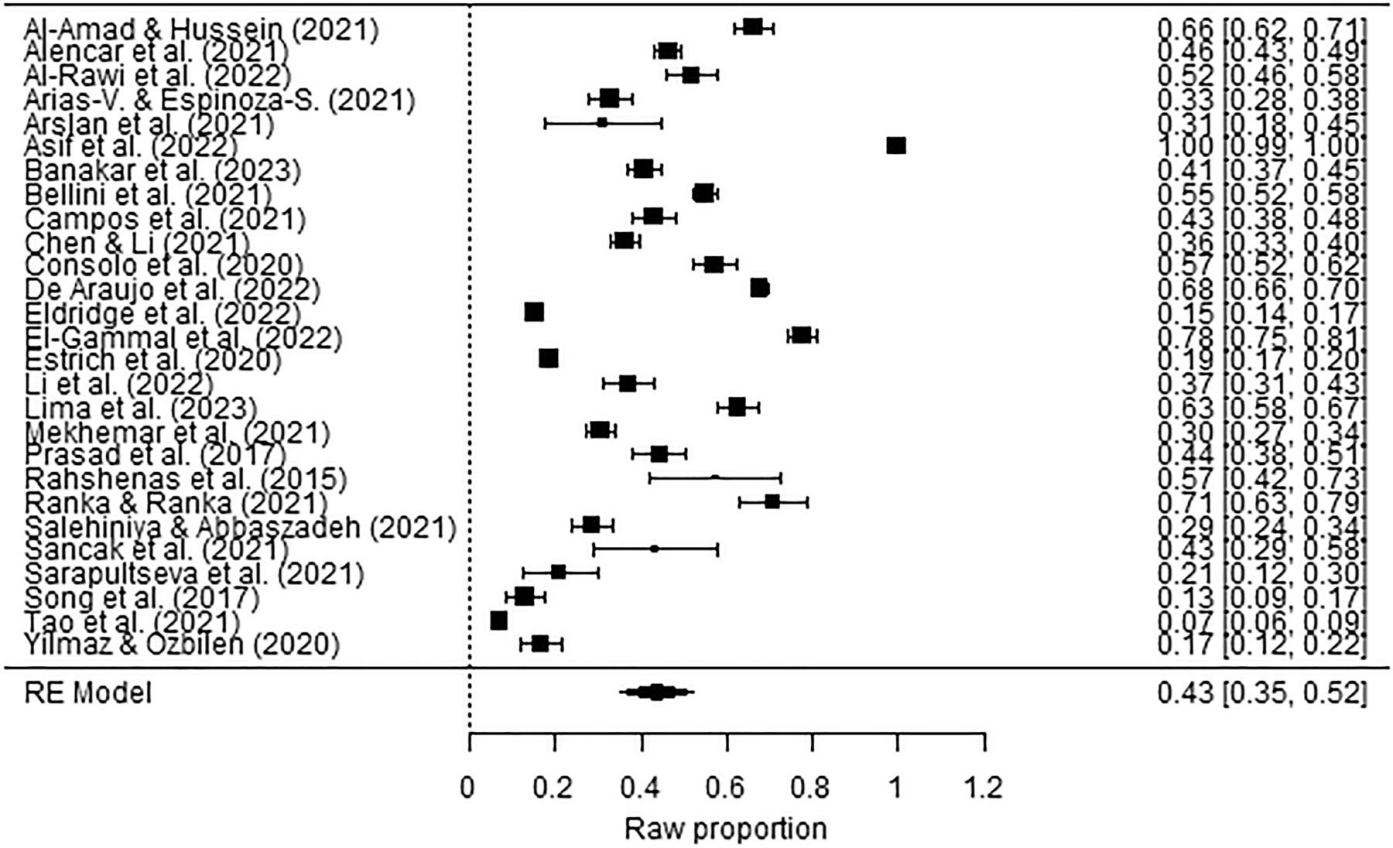
Forest plot–prevalence of anxiety symptoms among dentists (*k* = 27). The full surnames of the authors Arias‐Vásquez and Espinoza‐Salcedo are shortened in the figure due to technical reasons.

**Figure 4:**
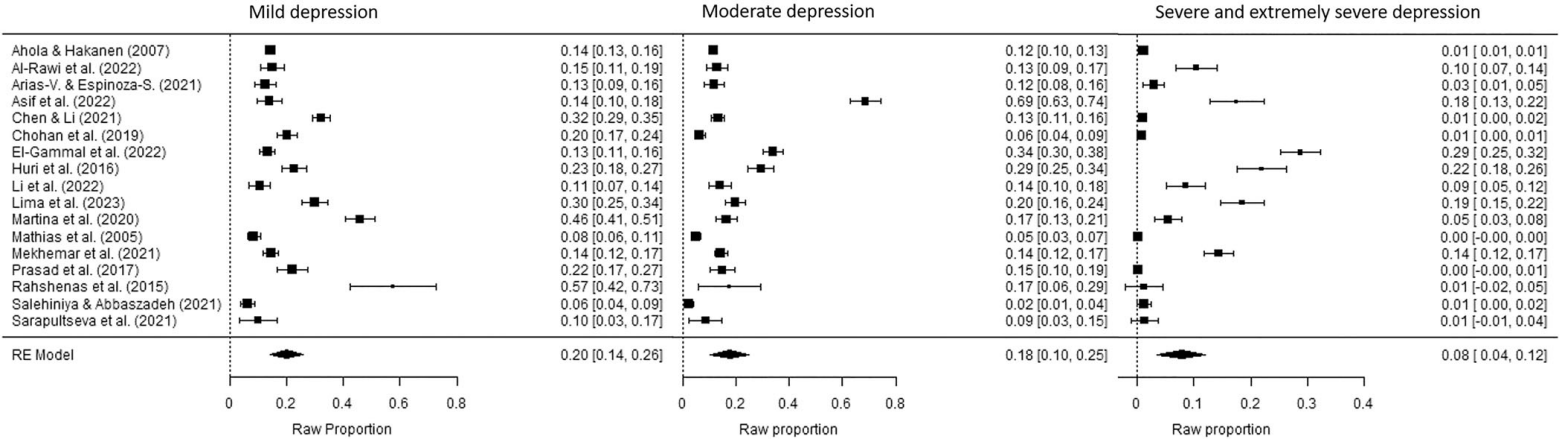
Forest plot–severity of depression symptom prevalence among dentists: mild, moderate, severe, and extremely severe (*k* = 17). The full surnames of the authors Arias‐Vásquez and Espinoza‐Salcedo are shortened in the figure due to technical reasons.

**Figure 5:**
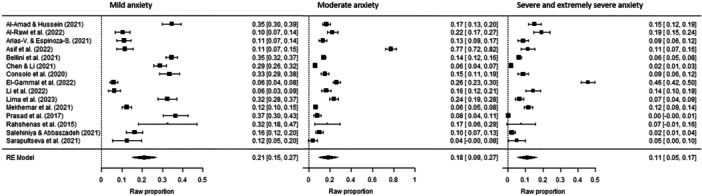
Forest plot–severity of anxiety symptom prevalence among dentists: mild, moderate, severe, and extremely severe (*k* = 15). The full surnames of the authors Arias‐Vásquez and Espinoza‐Salcedo are shortened in the figure due to technical reasons.

The prevalence of depression symptoms among dentists was 42% (*p* < 0.001, 95% CI [0.34, 0.51]), while the prevalence of anxiety symptoms was 44% (*p* < 0.001, 95% CI [0.35, 0.52]) (Figure 2 and Figure 3). The prevalence rates for different severity levels of depression symptoms among dentists were as follows: mild depression 20% (*p* < 0.001, 95% CI [0.14, 0.26]); moderate depression 18% (*p* < 0.001, 95% CI [0.10, 0.25]); and severe and extremely severe depression 8% (*p* < 0.001, 95% CI [0.04, 0.12]) (Figure 4). The prevalence rates for different severity levels of anxiety symptoms were as follows: mild anxiety 21% (*p* < 0.001, 95% CI [0.15, 0.27]); moderate anxiety 18% (*p* < 0.001, 95% CI [0.10, 0.27]); and severe and extremely severe anxiety 11% (*p* < 0.001, 95% CI [0.05, 0.17]) (Figure 5).

### 3.2 The Moderation Effect of the Time Period (Pre/During COVID) on the Prevalence of Depression and Anxiety Symptoms

The subgroup analysis, based on the time period (pre/during COVID), did not provide evidence of differences between groups in the prevalence of either depression or anxiety symptoms. Although the data suggest slightly higher prevalence rates of depression (10%) and anxiety (12%) among dentists before the COVID period, the observed differences (*p* = 0.32 for depression and *p* = 0.33 for anxiety) do not meet conventional thresholds to reject the null hypothesis. These findings indicate that the prevalence of depression and anxiety symptoms among dentists appears consistent across the two time periods analyzed.

### 3.3 Gender Differences in the Prevalence of Depression and Anxiety Symptoms

Statistically significant gender differences were found in the experience of depression (*p* < 0.001, 95% CI [0.09,0.38]) and anxiety symptoms among dentists (*p* = 0.03, 95% CI [0.03,0.40]). Women demonstrated an approximately 27% (95% CI [1.10, 1.46] higher risk of experiencing depression and 24% (95% CI [1.03, 1.50] higher risk of experiencing anxiety compared to men. This suggests higher prevalence rates of depression and anxiety symptoms among women dentists.

### 3.4 Publication Bias Examination

Publication bias tests and analyses were conducted separately for studies contributing to the determination of the overall prevalence of depression and anxiety symptoms, studies investigating the prevalence of symptom severity, and studies assessing gender differences in the prevalence of such symptoms among dentists.

The Fail‐safe N analyses, using the Rosenthal approach, was conducted to assess the robustness against potential inclusion of nonsignificant or unpublished studies. The results were significant at *p* < 0.001 for all analyses, indicating no evidence of publication bias.

Visual inspection of the funnel plots revealed some degree of asymmetry in all analysis. However, both the Rank correlation test and the Regression test for funnel plot asymmetry did not provide evidence of systematic asymmetry in the funnel plots for studies included in the gender difference analysis or those assessing the prevalence of severity of anxiety symptoms. Both tests yielded p‐values greater than 0.05, suggesting no statistical support for significant asymmetry.

Regarding the overall prevalence of depression and anxiety symptoms, we encountered mixed results. The Rank correlation test was significant for both depression and anxiety symptoms, whereas the Regression test for funnel plot asymmetry was nonsignificant for both. Considering the abovementioned outcome of fail‐safe N analysis, which indicated robustness against publication bias, it can be concluded that any possible systematic asymmetry in the funnel plot is not of major concern.

Finally, when examining funnel plot asymmetry regarding meta‐analysis results for various levels of depression symptoms, a clearer pattern was visible. While results for moderate depression did not provide evidence of systematic asymmetry, the analyses for mild, severe, and extremely severe depression symptoms indicated statistically significant asymmetry. In these cases, both the Rank correlation test and Regression test for funnel plot asymmetry were significant at *p* < 0.05. This finding suggests the potential presence of bias affecting the meta‐analysis results. Specifically, the observed effect size determined from this analysis might be inflated due to the potential exclusion of studies with nonsignificant results regarding analysis with mild, severe, and extremely severe depression symptoms.

## 4 Discussion

Our meta‐analysis showed high prevalence of depression and anxiety symptoms among dentists. A significant proportion of dentists had moderate to intense depression and anxiety symptoms.

It is generally accepted that dentistry is a stressful profession [45, 46]. The levels of self‐reported stress, burnout, and psychological stress in dentists are high, causing a serious concern [47]. Overall, the prevalence of mental health problems among health workers, including dentists, is higher than among general population [48].

The analysis did not provide evidence of differences in the prevalence of depression and anxiety symptoms among dentists before and during the COVID‐19 pandemic. This may suggest that dentists are vulnerable to stress due to the nature of dental practice, although the types of stressors were different in the pre‐pandemic and pandemic periods. Life stressors and psychological distress have been associated with the development of depression [49] and anxiety disorder, suggesting that the stressful nature of dental practice itself could be a contributing factor to dentist's mental problems [50].

The COVID‐19 pandemic has led to additional challenges for dentists in their everyday practice [45]. The pandemic has reduced their psychological resources, resulting in depression, fatigue, and burnout [16] and highlighting the importance of a tailored management response to their emotional demands at work [51].

Most studies included in this systematic review and meta‐analysis were conducted during the COVID‐19 pandemic; pre‐pandemic studies were of low quality (weak or invalid) and could not be included in the meta‐analysis to contribute to more objective conclusions. A systematic review of mental and physical health before pandemic period concluded that more prospective and retrospective research was needed, with studies using validated measuring instruments and statistically adequate analyses [52]. Interest in the impact of the COVID‐19 pandemic on the mental health and well‐being of frontline healthcare workers (HCWs), including dentists, has increased, because the pandemic has affected the deployment of significant resources, including HCWs, to mitigate the spread of disease and reduce morbidity and mortality [53]. The negative impact of the pandemic on dentists around the world has been confirmed, with fear of infection, family transmission, lack of personal protective equipment, and possible direct contact with infected patients being identified as key risk factors. Personal resilience and organizational support along with improved infection control protocols have been shown as important protective factors [54].

Among dentists, fear of infection and transmission were predictors of anxiety [6, 28], depression [1, 14], and both depression and anxiety [20, 23]. In general, the systematic review showed that the consequences of the COVID‐19 pandemic on dental professionals were psychological (anxiety, depression, fear, concern about the transmission of the disease to the family, stress, insomnia, and mental disorders) and professional (fear of job loss, financial worries, worries about insufficient health equipment, and worries about career prospects) [4]. Research has shown a deterioration in mental health and well‐being among dentists before the COVID‐19 pandemic [45, 46]. The identified stressors in dentistry were work environment, time constraints, risk and fear of litigation or regulatory fines, insurance contracts, unrealistically heavy workloads, and patient issues [46, 55]. Mental disorders, high levels of psychological distress, and burnout were reported among Australian dental practitioners, suggesting a need for education and support programs for mental health and well‐being [56].

There is some evidence that resilience protects against stress and mental health problems, highlighting the need for resilience training for healthcare workers, including dentists [57]. Still, a focus on resilience training may create the perception that mental health issues are an individual problem [58].

In our study, gender differences were observed in the prevalence of depression and anxiety symptoms, which was higher among female dentists. It seems that gender differences play an important role [2, 18] because female dentists are at greater risk of developing mental disorders than their male counterparts, although it remains unclear whether these findings are job‐specific. Women were found to have higher susceptibility, lower adaptation abilities, and lower resistance to mental stressors [59], as well as generally higher prevalence percentage of depression and anxiety [60]. Furthermore, the work‐related stress of dentists reflects directly on their family life, which is one more reason to tailor stress‐prevention strategies and measures. In addition, in certain cultures, women are under greater pressure related to their professional and family roles, which additionally leads to increased depressive and anxiety responses while they try to maintain a balance between professional and family life. Therefore, it is very important to pay attention to practice‐related disorders and their prevention.

This review confirmed the high level of depression and anxiety among dentists before and during pandemic. Any future initiatives should incorporate a longer‐term strategy that would support both mental and physical well‐being of dental professionals, given the stressfulness of their job [45, 46]. The distinction between “operational” and “organizational” elements may provide an important future framework for understanding the impact of job stressors in additional high‐stress healthcare professions [54].

## 5 Limitations

This study has several limitations. Firstly, the studies included in the meta‐analysis might not be fully representative of the population of dentists, limiting generalizability of the findings. Reporting bias is a common concern in meta‐analysis as studies with significant results are more likely to be published, potentially skewing the accuracy of the pooled estimates. Secondly, the included studies used a variety of methodologies to assess depression and anxiety, potentially leading to variability in results. Despite the use of a random‐effects model to account for this heterogeneity, the differences in study designs could still affect the validity of the conclusions drawn. Finally, the meta‐analysis did not take into account other potential moderating variables, such as geographic location, dental specialties, age, or education level, which could influence the prevalence rates of depression and anxiety among dentists.

The strengths of this study are inclusion of worldwide data, rigorous analysis of study quality, and substantial levels of concordance of reported clinical findings.

## 6 Contributions of the Study and Its Practical Implications

The study makes several significant contributions to the field. Firstly, the use of systematic review and meta‐analysis with the PRISMA protocol provides a more reliable and objective assessment of the prevalence and severity of depression and anxiety among dentists. Secondly, the study contributes to the theoretical understanding of mental health, focusing on dentists, a group often underrepresented in mental health research. The study adds to the existing literature and provides a basis for further exploration into occupational stress and mental health in specialized professions. By investigating a moderating effect of the COVID‐19 pandemic, our study acknowledges the impact of external, global events on professional mental health, offering a deeper understanding of the factors influencing mental health at workplace. Also, our study addresses gender differences among dentists, an area that has received limited attention in prior research. Our study fills a gap in the literature and highlights the significance of gender in the experience and manifestation of mental health challenges in high‐stress professions like dentistry.

The study offers several practical implications. Its findings, highlighting the high prevalence of depression and anxiety among dentists, particularly during the COVID‐19 pandemic, emphasize the need for mental health support programs and educational initiatives within the dental profession. Such initiatives could play a crucial role in reducing levels of depression and anxiety among dentists. These programs should aim to equip dentists with the tools and knowledge necessary to manage effectively the stress and mental health challenges, thereby fostering a healthier work environment and better patient care. Moreover, the study found a higher prevalence of mental health issues among female dentists, indicating the need for targeted interventions designed to address the unique challenges faced by female dentists in dentistry.

Finally, the results of the study clearly show that more research is needed in this area, especially regarding gender differences among dentists. Future studies should keep focusing on gender to better understand the reasons behind these differences in mental health issues between men and women.

## 7 Conclusion

The high level of depression and anxiety observed in dentists in period before and during the COVID‐19 pandemic, and a significant percentage of moderate to severe depression and anxiety, can negatively affect the mental health status of dentists and the amount and quality of dental services they provide. Furthermore, female dentists reported a higher prevalence of depression and anxiety than their male colleagues. Providing educational content to reduce their depression and anxiety will help to maintain the mental health status of dentists and help to provide better quality services. Future research on depression and anxiety associated with dentist‐specific and pandemic‐specific job stressors, including their complex interrelationship with recognizable risk and protective factors is suggested. This may provide an important future framework for understanding the impact of job stressors in additional high‐stress healthcare professions. Future interventions should take a holistic approach aimed at creating a healthy, safe, and supportive work environment, including organizational support from regulatory bodies and government along with continuous mental health monitoring.

## Author Contributions

**Zrnka Kovačić Petrović:** conceptualization, investigation, writing – original draft, methodology, data curation, validation, writing – review and editing, project administration, resources. **Tina Peraica:** conceptualization, investigation, funding acquisition, methodology, validation, writing – review and editing, data curation, resources, project administration. **Mirta Blažev:** writing – review and editing, visualization, methodology, formal analysis, software, data curation, investigation, funding acquisition, validation. **Vesna Barac Furtinger:** writing – review and editing, investigation, data curation, resources. **Dragica Kozarić‐Kovačić:** conceptualization, investigation, supervision, resources, project administration, methodology, writing – review and editing, validation, data curation.

## Ethics Statement

The authors have nothing to report.

## Conflicts of Interest

The authors declare no conflicts of interest.

## Transparency Statement

The lead author Tina Peraica affirms that this manuscript is an honest, accurate, and transparent account of the study being reported; that no important aspects of the study have been omitted; and that any discrepancies from the study as planned (and, if relevant, registered) have been explained.

## Data Availability

The data that support the findings of this study are available on request from the corresponding author. The data are not publicly available due to privacy or ethical restrictions.
